# Learning for doctor-to-doctor collaboration: a qualitative study exploring the experiences of residents and supervisors with intraprofessional workplace learning in complex tertiary care

**DOI:** 10.1186/s12909-023-04363-5

**Published:** 2023-06-27

**Authors:** Lara Teheux, Hanna Wollaars, Jos M.T. Draaisma, Ester H.A.J. Coolen, Wietske Kuijer-Siebelink, Janiëlle A.E.M. van der Velden

**Affiliations:** 1 Department of Pediatrics, Radboud University Medical Center, Radboudumc Amalia Children’s Hospital, Nijmegen, The Netherlands; 2 Department of Research on Learning and Education, Radboud University Medical Center, Radboudumc Health Academy, Nijmegen, The Netherlands; 3 School of Education, HAN University of Applied Sciences, Nijmegen, the Netherlands; 4 Department of Pediatrics, Radboud University Medical Center, Amalia Children’s Hospital, Nijmegen, The Netherlands

**Keywords:** Intraprofessional learning, Collaborative practice, Postgraduate medical education, Workplace learning

## Abstract

**Background:**

To deliver high-quality care for individuals with complex medical conditions, residents need to be trained across the boundaries of their specialties. This study aimed to explore learning activities and influencing factors in intraprofessional workplace learning by residents in complex tertiary care.

**Methods:**

This qualitative study was conducted in a tertiary care children’s hospital. In September – December 2017, fourteen individual and two focus group interviews were conducted with a purposive sample of residents and supervisors of various specialties. Transcribed interviews were thematically analyzed to describe learning activities and influencing factors that play a role in intraprofessional workplace learning in complex tertiary care settings during residency training.

**Results:**

Respondents described numerous activities that they considered opportunities for intraprofessional learning, both directly and not directly related to patient care. However, deliberate attention to intraprofessional learning often seemed to be lacking in clinical practice. Influencing factors on a system (macro), organization (meso) and personal and interpersonal level (micro) level were identified. Factors on the macro and meso level mainly determined whether intraprofessional learning opportunities arose, while micro level factors mainly influenced whether opportunities were seized.

**Conclusions:**

There are ample opportunities for intraprofessional workplace learning in complex tertiary care for residents. Residents may benefit more from intraprofessional learning opportunities if these are made more intentional and deliberate. Influencing factors at the macro, meso and micro level provide targets for interventions aimed at enhancing intraprofessional workplace learning in postgraduate medical training.

**Supplementary Information:**

The online version contains supplementary material available at 10.1186/s12909-023-04363-5.

## Background

Healthcare professionals are faced with increasingly complex health issues that require professionals from different backgrounds to collaborate effectively in order to provide high-quality patient care [1, 2]. The increasing specialization within the medical profession imposes a significant threat to patient care as it may lead to fragmentation in disciplinary silos and ineffective collaboration, which are notorious causes for harmful medical errors [2–4]. Hence, medical trainees need to be trained across the boundaries of their specialties to learn the skills required for collaborative care [1, 5, 6]. This is of particular importance in complex tertiary care, where care is provided for individuals with highly complex care needs who often require collaborative care from multiple highly specialized health professionals [7–9].

Gaining an understanding of factors that influence intraprofessional workplace learning (i.e., the learning that occurs when individuals of two or more disciplines within the same profession engage in workplace-related activities [5]) by residents in tertiary care settings is vital towards creating a positive impact on intraprofessional learning and collaboration for complex care. A recent scoping review established intraprofessional learning outcomes and a multitude of factors that influence intraprofessional workplace learning [10]. However, few studies included in this review examined intraprofessional learning in complex tertiary care, and those that did were restricted to the perspective of one specialty or investigated a singular training. None of these studies considered intraprofessional workplace learning in the broader context of the diversity of specialties involved in a tertiary care center. It seems likely that the highly complex nature of care processes in tertiary care brings forth specific opportunities and challenges that warrant further investigation of intraprofessional workplace learning in this context.

Residents need to interact with a diversity of specialties throughout their learning trajectory to become ‘collaborative practice-ready’ health professionals for complex care [6, 11]. Hence, we argue that intraprofessional learning in residency training in tertiary care should not be viewed through the narrow lens of dyadic interaction between specialties, but rather from the broader perspective of the multiple specialties brought together in a tertiary care center. Therefore, unravelling the factors that influence intraprofessional workplace learning in a tertiary care center constitutes a crucial step towards achieving meaningful intraprofessional learning trajectories for complex patient care. This study set out to advance understanding of intraprofessional workplace learning by residents in complex tertiary care, aiming to support the design of meaningful intraprofessional clinical learning environments. The research questions of this study were: (1) What intraprofessional learning activities do residents and supervisors experience at the workplace in a tertiary care center? and (2) What are the factors that influence intraprofessional workplace learning by residents in a tertiary care center?

## Methods

### Study design

Starting from a constructivist research paradigm, we designed a qualitative interview study, as a qualitative approach was considered appropriate for exploring the complex dynamics of intraprofessional workplace learning in tertiary care [12]. We aimed to capture common features and influencing factors involved in intraprofessional workplace learning through individual and focus group interviews with residents and supervisors. The reporting of this study was guided by the Standards for Reporting Qualitative Research [13]. (See Additional File 1).

### Context

This research was conducted at the Radboudumc Amalia Children’s Hospital, a tertiary pediatric care center in the Netherlands where approximately 22,000 children are treated annually. The Radboudumc Amalia Children’s Hospital consists of two medium care departments with 48 beds, a short stay medium department with 10 beds, a pediatric intensive care and high care department with 8 beds, a neonatal intensive, high and medium care department with 35 beds, and an outpatient clinic and an emergency department. In the Radboudumc Amalia Children’s Hospital, doctors from 23 different specialties work together. Training residents is an integral part of the work environment and residency training programs are coordinated separately for each specialty.

### Data collection

This study included a purposive sample of residents and supervisors of various medical specialties. We decided to study intraprofessional workplace learning in postgraduate training from the experience of both residents and supervisors as the dyadic interaction between residents and their supervisors is at the core of postgraduate training [14]. All residents and supervisors from the Radboudumc Amalia Children’s Hospital that were not involved in the research project were eligible for participation. Participants were invited for participation by an independent party, namely the secretarial office. A purposive sampling strategy was used to select participants from different surgical, non-surgical and supportive specialties with varying levels of involvement in complex tertiary child care in order to collect experiences with intraprofessional workplace learning from diverse perspectives (Table 1) [12]. Due to the rotation of residents, participants were able to report from a broader range of experiences in various departments.

**Table 1:** Study participants

Interview	Role	Specialty
Individual Interviews	Residents	Anesthesiology (R1), dermatology (R2), general surgery (R3), otorhinolaryngology (R4), pediatrics (R5), radiology (R6), pathology (R7), urology (R8).
	Supervisors	Intensive care medicine (S1), neurology (S2), ophthalmology (S3), gynecology and obstetrics (S4), neurosurgery (S5), rehabilitation medicine (S6).
Focus group interview 1	Residents	Plastic surgery (R9), oral and maxillofacial surgery (R10).
	Supervisors	Orthopedic surgery (S7), radiology (S8), psychiatry (S9).
Focus group interview 2	Residents	Rehabilitation medicine (R11), human genetics (R12).
	Supervisors	Dermatology (S10), pediatric surgery (S11), pediatrics (S12), emergency medicine (S13), urology (S14).

Semi-structured individual interviews were conducted with eight residents and six supervisors. The interview guide was developed by a project group of educationalist, supervisors and residents and is provided in Additional file 2. Prior to the interview, the phenomenon of intraprofessional learning was explained by the interviewer to ensure a common understanding of this concept.

Next, two focus group interviews with different respondents were conducted to promote data triangulation and to further enrich and deepen the findings from the individual interviews through an interactive discussion between group members. Heterogenic focus groups were purposively formed to stimulate a discussion from different perspectives. The themes from the individual interviews were presented at the beginning of the focus groups and used as a starting point for the discussions. The guiding questions for the focus group interview are provided in Additional File 3. Two moderators led the focus group: one serving as main moderator who directed the flow of the conversation, and the other as an observer who focused on participants’ responses and asking follow-up questions that the main operator might have missed due to their moderating duties.

The interviews and focus groups were conducted by two educationalists who were trained and experienced in qualitative research and conducting interviews and focus groups. The participants and interviewers did not know each other. All interviews were audiotaped, transcribed verbatim and anonymized.

Data collection was ended when the project group established they collected sufficient data to meet the project goals [15]. Data collection was performed September through December 2017.

### Data analysis

Data was analyzed using thematic analysis according to Braun and Clarke, following six steps: (1) familiarizing oneself with the dataset, (2) generating initial codes, (3) identifying themes, (4) reviewing themes, (5) defining and naming themes, and (6) producing the report [16]. The data analysis was performed in a research team with diverse backgrounds. Three researchers (JD,EC,JV) were ‘insiders’ in the institution (i.e., working as medical specialist within the studied clinical learning environment) and, therefore, able to interpret findings from the local sociocultural context. The other three researchers (LT,HW,WK) could act as ‘outsiders’ and question notions that otherwise might have been taken for granted, such as notions regarding organization of work or collaborative practices. Explicitly reflecting on and discussing our ‘insider’ and ‘outsider’ perspectives throughout the research process contributed to the practice of reflexivity. Before analysis, the researchers explicitly formulated and discussed their own assumptions about the studied phenomenon. Keeping a reflective journal and engaging in discussions about personal assumptions in relation to the research data promoted reflexivity and confirmability.

Atlas.ti (v8.4.20) was used to organize the data. In phase one, we familiarized ourselves with the data by reading and rereading the transcripts. In phase two, transcripts from the individual and focus group interviews were coded by two researchers (LT,HW) using an inductive approach. Differences in coding were solved by discussion. A third researcher (JV) was involved in case further discussion was necessary. In phase three, the research team (LT,HW,JD,EC,WK,JV) discussed the codes and categorized them in preliminary (sub)themes. While reviewing the themes in phase four, the research team felt that clustering the influencing factors in the system (macro), organization (meso), and personal and interpersonal (micro) level would be helpful as an interpretive tool to support educational practice by giving readers an understanding of the factors that could be addressed by the respective parties at each of these levels. Inspired by the framework of “Interprofessional Education for Collaborative Patient-Centred Practice” (IECPCP) [17, 18], the research team operationalized the respective levels as follows: macro (system) level factors relate to the wider sociocultural environment and are beyond the direct influence of the organization and its individuals; meso (organizational) level factors relate to the organizational setting, including the organization of the work and learning environment; and micro (personal and interpersonal) level factors relate to the individuals involved and their interactions and relationships. LT and HW reviewed the (sub)themes and underlying codes and data extracts to ensure that the categorization of themes and subthemes was consistent and reflected the essence of the underlying data. In phase five, the research team refined the names of every theme and subtheme to ensure that it captured its underlying essence accurately. Finally, in phase six, the research team conducted a critical review of the manuscript to ensure that the identified themes were accurately represented. This final review process ensured that the manuscript presented a clear and comprehensive account of the study’s findings.

### Ethical considerations

This research was performed in accordance with the Declaration of Helsinki. The Radboudumc Research Ethics Committee (IRB) approved this study (file number 2020–6284). Participants were informed of their rights, the aims of this study and how their data is protected. Informed consent was obtained from all participants.

## Results

This study investigated (1) what intraprofessional workplace learning activities are experienced by residents and supervisors and (2) what factors influence intraprofessional workplace learning. In general, participants expressed a positive attitude towards intraprofessional learning. They considered intraprofessional learning *“educational”* (R8) and “*super interesting”* (S3), and their experiences with intraprofessional workplace learning as *“valuable”* (R6; R7; S9). Respondents believed that intraprofessional learning improves medical knowledge and collaboration between specialties and, consequently, patient care.

**Fig. 1:**
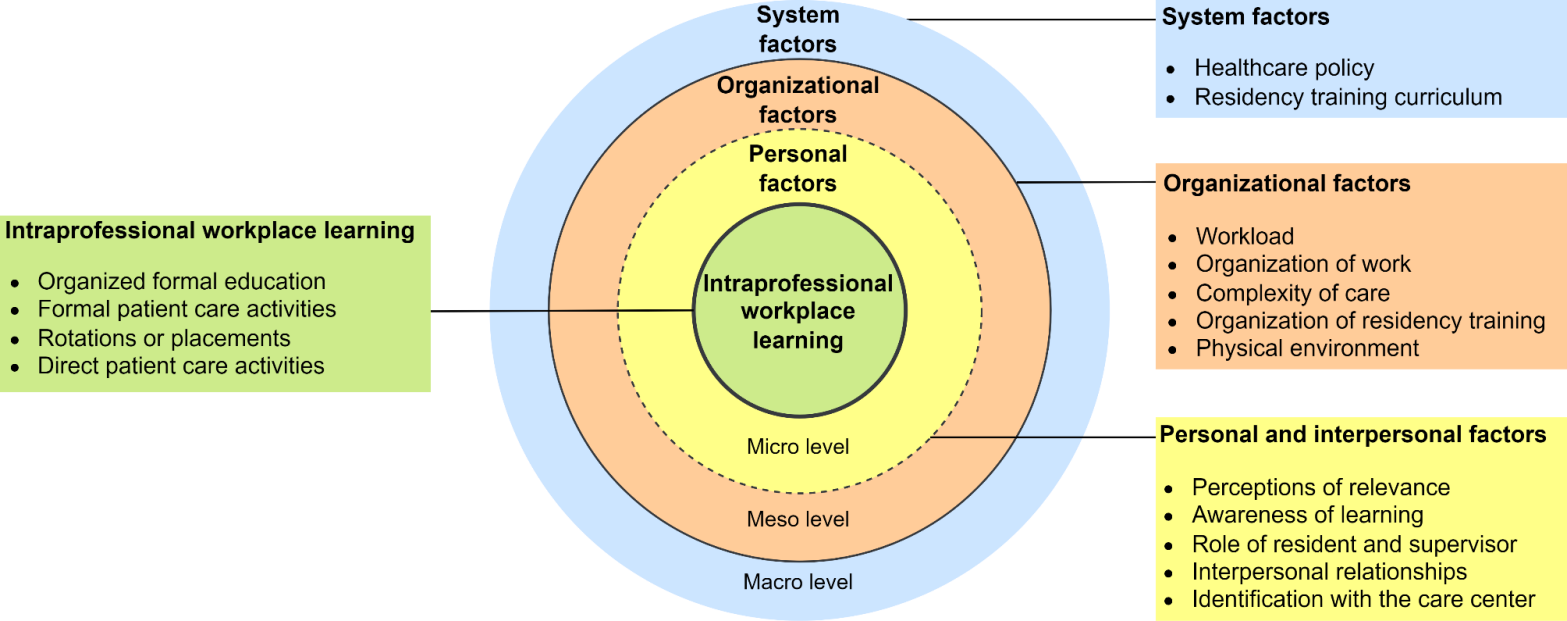
Visual summary of results

The results will first address the learning activities experienced by residents and supervisors. Second, the influencing factors on the system (macro), organization (meso) and personal and interpersonal (micro) level will be reported. The results are visually represented in Fig. 1.

### Learning activities

Respondents identified intraprofessional learning activities they experienced at the workplace, which we organized in four categories: organized formal education, formal patient care activities, rotations or placements and direct patient care activities (Table 2).

**Table 2:** Reported learning activities summarized in four main categories

Category	Description
Organized formal education	Organized, formal educational activities involving doctors from two or more specialties. Both attending and delivering formal education were mentioned as learning opportunities.
Formal patient care activities	Formal meetings or activities directly related to patient care, such as multidisciplinary meetings (MDMs) and intraprofessional handovers.
Rotations or placements	Intraprofessional rotations or placements in which the learner participates in or shadows the care activities of another specialty.
Direct patient care	Providing patient care together with someone from another specialty, e.g., shared care on the inpatient wards or in the emergency department, inpatient consultations, or joint consultation hours in the outpatient clinic.

Respondents frequently commented on existing formal educational activities in which residents from two or more specialties were involved, which were organized by one specialty for another or for multiple specialties together. Participants envisioned possibilities to organize new intraprofessional formal education or to exploit already existing uniprofessional education:

*“You could say, well, if there are some neurological subjects, I will ask the neurology resident to join.”* (R5).

Formal patient care activities, such as multidisciplinary meetings (MDMs) were considered valuable intraprofessional learning opportunities, although respondents commented that these could be better utilized by assigning residents a more active role:

*“The MDMs I attend are predominantly the responsibility of the staff members. … That’s a shame, because when you prepare for an MDM, you’re not only required to immerse yourself in a patient, but also to present your colleagues with everything they want to hear. However, since these MDMs are attended by a relatively small number of residents from other disciplines as well – and when they do attend, they have a seat in second row and only introduce a patient now and then – I absolutely believe that there are serious opportunities to expand on this.”* (S8).

Another frequently mentioned learning opportunity was shadowing other specialties or intraprofessional rotations. These were considered highly valuable for both parties, since it provides a better understanding of each other’s perspectives and working environment and *“also gives an enormous boost to the relationship between individual specialties”* (S8). A supervisor emphasized how placements are educational for both parties because *“the exchange that arises is so valuable”* (S9). Participants suggested that both residents and supervisors should be given more time to shadow other specialties, as this was considered a highly valuable learning opportunity.

Respondents experienced that intraprofessional learning could arise from providing patient care together with someone from another specialty: in the emergency department, in joint consultation hours in the outpatient clinic or in intraprofessional consultations concerning a patient on the ward. Furthermore, examples were given of departments where residents of multiple specialties directly work together, which allowed residents to learn from each other’s expertise in the shared patient care:

*“Ventilation equipment is a piece of cake for anesthesiology assistant physicians, but this absolutely doesn’t apply to pediatric assistant physicians. And then you see that they’re training each other at a certain moment.”* (S1).

Supervisors argued that intraprofessional learning directly related to patient care in the workplace was more instructive, because “*the way of learning for most doctors is to actually do something”* (S9).

During these activities, residents did not only learn from the perspectives and knowledge of other specialties, but residents also reported learning about intraprofessional care from their own supervisors when discussing or observing intraprofessional collaboration because “*then you start to see how they seek that collaboration”* (R12).

### Influencing factors

A multitude of system (macro), organization (meso) and personal and interpersonal (micro) level factors that influence intraprofessional workplace learning in complex tertiary care were derived from the interviews (Fig. 1). Overall, factors at the system and organization level mainly seemed to determine whether intraprofessional learning opportunities arose, while personal and interpersonal level factors influenced whether these opportunities were seized.

#### System factors (macro level)

The learning opportunities to which residents are exposed are influenced by the organization of health care, training curricula and workforce planning of residency training. Respondents felt that training curricula seemed to mainly concern their own specialty, instead of crossing boundaries. They suggested that the explicit inclusion of intraprofessional learning in curricula might help secure sufficient time for intraprofessional activities and might stimulate learners and supervisors to seek intraprofessional learning opportunities. For example:

*“You could make it part of those EPAs. …That will also stimulate them more: I have to do something with that as well.”* (S14).

#### Organizational factors (meso level)

A frequently recurring barrier was a lack of time and high workload. Respondents argued that intraprofessional learning activities *“[need] to be embedded practically in such a way so that it won’t feel as yet another thing we have to do on top of everything else”* (R5). Too much focus on clinical productivity was considered a barrier, as this resulted in a diminished focus on learning. Furthermore, respondents experienced that learning opportunities were missed due to malalignments in the organization of work between specialist departments, for example due to differences in logistics and planning, and a lack of insight in each other’s working schedule.

Although the high volume of patient cases involving multiple specialties in tertiary care was considered a learning opportunity, the high complexity of tertiary care was experienced as a barrier because it hindered residents to actively participate in intraprofessional care:

*“It’s not without reason that patients are discussed during a multidisciplinary meeting. Therefore, it can be very complicated. And there comes a moment when you, as a resident, tune out.”* (R5).

*“Since these are often highly complicated children, the medical specialists often are the ones doing the talking during these meetings.”* (S6).

Intraprofessional learning was also influenced by how the residency training was organized at the workplace. Interviewees experienced multiple difficulties specifically related to the organization of intraprofessional formal education, such as logistical challenges, sufficient overlap in subjects and perceived need for comparable knowledge on these subjects beforehand.

An enabling physical environment was an important prerequisite for intraprofessional workplace learning, e.g., enough physical space in consultation rooms. Respondents noted that physical proximity between specialties makes it easier to interact. For example, a shared physicians’ room augmented intraprofessional interaction:

*“It’s much easier to find each other for a consultation. You are there, the short lines of communication are there, you know each other better, you talk to each other more easily.”* (S13).

#### Personal and interpersonal factors (micro level)

In order to be open to intraprofessional learning, it seemed crucial that residents and supervisors feel that it is worth their investment:

*“When you think about intraprofessional training, it is only useful to do it with specialties that are actually involved in the problem.”* (R4).

Furthermore, respondents felt that many intraprofessional activities were a learning opportunity but that these were neither recognized nor utilized as such. For example, respondents felt that multidisciplinary meetings were often conducted with a sole focus on medical aspects of patient care and *“could be further elaborated, or … at least be identified more actively as learning moments”* (R9).

Respondents said that what residents learn from intraprofessional learning activities partly depends on the extent to which they have an active role; you learn more *“if you do more things yourself”* (S6) or “*when you bear responsibility for things”* (S8). If residents have an active role depended on both the residents’ personal interest and the opportunities to actively participate that they were provided by the organization of work or their supervisors. Multiple reasons for supervisors to bypass residents came forward, including high workload, the perception that it is better for clinical productivity, the high complexity of care, and the fact that some intraprofessional activities are unplanned.

*“I’ve noticed that other specialties also don’t come with a resident when they visit. … They simply drop in and ask questions. This is the fastest and easiest way for clinical productivity, naturally.”* (R1).

Interpersonal relationships were considered important for intraprofessional workplace learning because participants felt that it was easier to consult someone they knew personally. Peer-contact with residents with a comparable level of experience was considered an especially safe learning environment, because *“it’s nice to have someone to spar with about something you both still don’t know that much about yet”* (R6). One supervisor mentioned a conflict between two specialties, which was detrimental to their relationship and, consequently, to intraprofessional learning.

Furthermore, the feeling of belonging to the tertiary care center was reported to influence learning; residents who felt less connected to the center expressed less need for intraprofessional activities. Positive experiences during an internship could provide a stronger connection.

## Discussion

Intraprofessional learning is vital towards achieving high-quality collaborative care for individuals with complex care needs. In this study, we identified learning activities and factors that influence intraprofessional workplace learning in complex tertiary care from the perspective of residents and clinical supervisors.

Respondents described numerous activities that they considered opportunities for intraprofessional learning. However, when the research team reflected upon the accounts, we noted that during many of these activities no deliberate attention seemed to have been paid to intraprofessional learning, and that intraprofessional learning merely seemed to be a byproduct of clinical care activities. Previous studies have also observed this lack of awareness [19–21]. Although the tacit nature does not exclude the occurrence of meaningful learning [22–24], learning opportunities are probably best utilized when learning is made more explicit and intentional [25, 26]. This improves individual and team performance through feedback, enables transfer of knowledge between individuals, increases accountability and leads to the construction of artefacts that can assist in decision-making and reasoning [25]. Moreover, implicit learning may enforce problematic stereotyping and impede care innovations [26]. Several accounts in the current study reflected a need to make intraprofessional learning more intentional and explicit. Since it can be difficult to learn how to deal with highly complex care challenges without an explicit interactive process of learning with other professionals [22], we propose that making intraprofessional learning more deliberate could be of particular importance in complex tertiary care.

We found numerous factors that influence intraprofessional workplace learning. Interestingly, macro and meso level factors mainly seemed to determine whether intraprofessional learning opportunities emerged, while micro level factors influenced whether other health professionals provided space for learning and whether learners elected to utilize these opportunities. This finding can be understood through Billet’s conceptualization of workplaces as learning environments; participation in workplace learning is constructed through interdependent processes of “workplace affordances” and “learner agency”, i.e., the opportunities to participate in learning practices afforded to learners by the workplace and its workers, and learners’ agentic behavior [22].

Surprisingly, despite the interview’s focus on individuals’ experiences with intraprofessional learning within the clinical environment, respondents frequently commented on influencing factors that were beyond their direct influence and that of the organization, such as healthcare policy and the national residency training curriculum. This finding highlights that system factors influence affordances within the clinical learning environment.

The influencing factors found in this study are similar to those reported in other studies conducted in other contexts, e.g., system factors, workload, organization of work, interpersonal relationships, motivation, and awareness of learning [10, 19, 27-29]. Moreover, this study found several factors that seemed particularly relevant to intraprofessional workplace learning in the tertiary care setting.

Previous literature has predominantly depicted complex care as a highly potent avenue for learning where doctors from different specialties interact [20, 30, 31]. However, the current study shows that too much complexity can be detrimental to residents’ learning, because without proper guidance the high complexity could lead to residents becoming disengaged and bypassed by their supervisors. Providing guidance is challenging because it is a continuous balancing act between enabling trainee autonomy and providing support, and it should encompass both the clinical tasks (e.g., observing, giving instructions) as well as the learning process itself (e.g., critical reflection, discussion) [32]. Hence, training supervisors may be a key step towards making optimal use of the learning potential of complex intraprofessional care.

Consistent with previous studies, this study illustrates that physical distance and logistics are key factors that determine which specialties interact and how this interaction takes place [33, 34]. Therefore, it is advisable to take into account intraprofessional learning when hospital layouts and logistical processes are being designed, so that residents from different specialties can interact at the workplace.

In line with other studies, we found that interpersonal relationships affect intraprofessional learning [19, 29, 30, 33-35]. This study adds to our understanding that a feeling of connectedness to the wider tertiary care center also affects the significance trainees attach to intraprofessional learning.

### Implications for practice

The influencing factors reported in this study provide a starting point for targeted interventions that can be taken to enhance intraprofessional workplace learning in complex tertiary care. Table 3 summarizes recommendations for clinical practice derived from our findings.

It is our recommendation that both workplace affordances and learner agency should be considered in the design of intraprofessional learning environments. Furthermore, the incorporation of intraprofessional learning in the training and assessment objectives of residency curricula might not only expand workplace affordances, but also provide an incentive for supervisors and residents to deliberately engage in intraprofessional workplace learning. As macro level factors affect affordances within the clinical learning environment, we recommend that intraprofessional learning should be considered in the design of health care policy and training curricula.

The respondents’ accounts reflected that in daily clinical practice there is a lack of deliberate attention to intraprofessional learning. We propose that a high priority should be given in particular to efforts aimed at ensuring high-quality guidance in complex care and at making intraprofessional learning a deliberate practice. Potential strategies for this include devoting explicit attention to learning in existing intraprofessional activities such as MDMs, training health professionals to facilitate the learning of others during these activities, and allocating time for individual and team reflection on intraprofessional care processes [10, 26, 29, 36]. Individual and team reflective practice is particularly important when health professionals engage in complex tasks such as in intraprofessional care [36], and it is a skill that requires explicit training of residents and facilitators [37].

Lastly, this study found that the feeling of connectedness affects how residents feel about learning intraprofessionally. This raises the possibility that agentic behavior could be stimulated through fostering meaningful intraprofessional relationships.

**Table 3:** Findings and practical recommendations

Findings from our study	Recommendations for practice	
Macro and meso level factors determined whether learning opportunities emerge, while micro level factors influenced whether learning opportunities are utilized.*	Consider both workplace affordances and learner agency [22] in the design of intraprofessional learning environments. Incorporate intraprofessional learning in training and assessment objectives, as this might expand workplace affordances, as well as provide an incentive for deliberate engagement by residents and supervisors.	
Health care policy and national training curricula influence intraprofessional learning in the clinical environment.	Design healthcare policy and training curricula in collaboration with clinical staff in order to support intraprofessional learning.	
Physical distance and logistics determine which specialties interact and how this interaction takes place.	Take into account intraprofessional learning when designing hospital layouts and logistical processes.	
Lack of deliberate attention to intraprofessional learning.	Devote explicit attention to learning in existing intraprofessional activities. Allocate time for individual and team reflection on intraprofessional care processes [36] Train health professionals to facilitate the learning of others [37]	
Too much complexity of care can be detrimental to residents’ learning.	Train supervisors how to recognize learning opportunities and how to provide guidance in complex care [32, 37]	
Interpersonal relationships and the feeling of connectedness to the care center affect intraprofessional learning.	Foster meaningful intraprofessional relationships to stimulate learner agentic behavior.	

* Macro (system) level factors relate to the wider sociocultural environment and are beyond the direct influence of the organization and its individuals; meso (organizational) level factors relate to the organizational setting, including the organization of the work and learning environment; and micro (personal and interpersonal) level factors relate to the individuals involved and their interactions and relationships.

### Strengths and limitations

To our knowledge, this is the first study to examine intraprofessional workplace learning from the perspective of the diversity of specialties involved in a tertiary care center. This study included perspectives from both residents and supervisors, and from various medical specialties. Rigor was promoted in this study by data triangulation using individual and focus group interviews, and by stimulating reflexivity in a research group that included insider and outsider perspectives.

While this study has provided valuable insights into residents’ and supervisors’ experiences with intraprofessional workplace learning and their reasoning based on these experiences, the interviews did not explore participants’ lived experience in depth. Furthermore, it is important to acknowledge that interviews are limited with respect to eliciting more tacit aspects. Field research could help elucidate hidden aspects that play a role in intraprofessional workplace learning. Second, the single center nature of this study might limit the transferability of its results. However, considering that the results of this study are consistent with previous studies in different contexts, we consider it probable that the lessons from this study are transferable to other contexts.

## Conclusions

Complex tertiary care offers ample opportunities for intraprofessional learning at the workplace, but deliberate attention is often lacking. Influencing factors at the system (macro), organization (meso) and personal and interpersonal (micro) level provide targets for interventions aimed at enhancing intraprofessional workplace learning in residency training for complex medical care.

## Electronic supplementary material

Below is the link to the electronic supplementary material.

Supplementary Material 1

Supplementary Material 2

Supplementary Material 3

## Data Availability

The dataset used and analyzed during the current study are available from the corresponding author upon reasonable request.
