# Supplementary material for: Learning for doctor-to-doctor collaboration: a qualitative study exploring the experiences of residents and supervisors with intraprofessional workplace learning in complex tertiary care
Source: BMC Med Educ. 2023 Jun 27;23:478. doi: 10.1186/s12909-023-04363-5 (PMC10303293; doi:10.1186/s12909-023-04363-5)
Supplement: Supplementary file 1 — Supplementary Material 1 [file 12909_2023_4363_MOESM1_ESM.pdf]

Additional file 1: filled SRQR checklist

Learning for doctor-to-doctor collaboration: a qualitative study exploring the experiences of residents and supervisors with intraprofessional workplace learning in complex tertiary care

BMC Medical Education

Lara Teheux, Hanna Wollaars, Jos M.T. Draaisma, Ester H.A.J. Coolen, Wietske Kuijer-Siebelink, Janiëlle A.E.M. van der Velden.

Corresponding author

Lara Teheux, MD, PhD-student, Department of Pediatrics, Amalia Children's Hospital, Radboud University Medical Center, Nijmegen, The Netherlands.

E-mail: lara.teheux@radboudumc.nl

Standards for Reporting Qualitative Research (SRQR)*

http://www.equator-network.org/reporting-guidelines/srqr/

Page/line no(s).

Title and abstract

**Title** - Concise description of the nature and topic of the study Identifying the study as qualitative or indicating the approach (e.g., ethnography, grounded theory) or data collection methods (e.g., interview, focus group) is recommended	Page 1 Line 3-5
**Abstract** - Summary of key elements of the study using the abstract format of the intended publication; typically includes background, purpose, methods, results, and conclusions	Page 2-3 Line 38-67

Introduction

**Problem formulation** - Description and significance of the problem/phenomenon studied; review of relevant theory and empirical work; problem statement	Page 4-5 Line 71-107
**Purpose or research questio**n - Purpose of the study and specific objectives or questions	Page 5 Line 101-107

Methods

**Qualitative approach and research paradigm** - Qualitative approach (e.g., ethnography, grounded theory, case study, phenomenology, narrative research) and guiding theory if appropriate; identifying the research paradigm (e.g., postpositivist, constructivist/ interpretivist) is also recommended; rationale**	Page 5 Line 110-115
**Researcher characteristics and reflexivity** - Researchers' characteristics that may influence the research, including personal attributes, qualifications/experience, relationship with participants, assumptions, and/or presuppositions; potential or actual interaction between researchers' characteristics and the research questions, approach, methods, results, and/or transferability	Page 7 Line 157-158 Page 8 Line 172-178 Page 9 Line 191-194 Page 22-23 Line 496-510
**Context** - Setting/site and salient contextual factors; rationale**	Page 5-6 Line 117-126
**Sampling strategy** - How and why research participants, documents, or events were selected; criteria for deciding when no further sampling was necessary (e.g., sampling saturation); rationale**	Page 6 Line 129-138 Page 8 Line 161-162
**Ethical issues pertaining to human subjects** - Documentation of approval by an appropriate ethics review board and participant consent, or explanation for lack thereof; other confidentiality and data security issues	Page 9 Line 196-200
**Data collection methods** - Types of data collected; details of data collection procedures including (as appropriate) start and stop dates of data collection and analysis, iterative process, triangulation of sources/methods, and modification of procedures in response to evolving study findings; rationale**	Page 6-8 Line 128-163

**Data collection instruments and technologies** - Description of instruments (e.g., interview guides, questionnaires) and devices (e.g., audio recorders) used for data collection; if/how the instrument(s) changed over the course of the study	Additional file 2<br>Additional file 3
**Units of study** - Number and relevant characteristics of participants, documents, or events included in the study; level of participation (could be reported in results)	Table 1
**Data processing** - Methods for processing data prior to and during analysis, including transcription, data entry, data management and security, verification of data integrity, data coding, and anonymization/de-identification of excerpts	Page 8<br>Line 159-160
**Data analysis** - Process by which inferences, themes, etc., were identified and developed, including the researchers involved in data analysis; usually references a specific paradigm or approach; rationale**	Page 8-9<br>Line 165-194
**Techniques to enhance trustworthiness** - Techniques to enhance trustworthiness and credibility of data analysis (e.g., member checking, audit trail, triangulation); rationale**	Page 7<br>Line 147-149<br>Page 7-8<br>Line 153-159<br>Page 8<br>Line 171-178<br>Page 9<br>Line 191-194

Results/findings

**Synthesis and interpretation** - Main findings (e.g., interpretations, inferences, and themes); might include development of a theory or model, or integration with prior research or theory	Page 9-16<br>Line 202-345<br>Figure 1<br>Table 2
**Links to empirical data** - Evidence (e.g., quotes, field notes, text excerpts, photographs) to substantiate analytic findings	Page 9-16<br>Line 202-345

Discussion

**Integration with prior work, implications, transferability, and contribution(s) to the field -** Short summary of main findings; explanation of how findings and conclusions connect to, support, elaborate on, or challenge conclusions of earlier scholarship; discussion of scope of application/generalizability; identification of unique contribution(s) to scholarship in a discipline or field	Page 16-21<br>Line 347-462<br>Table 3
**Limitations** - Trustworthiness and limitations of findings	Page 20-21<br>Line 440-455

Other

**Conflicts of interest** - Potential sources of influence or perceived influence on study conduct and conclusions; how these were managed	Page 21<br>Line 477-478
**Funding** - Sources of funding and other support; role of funders in data collection, interpretation, and reporting	Page 22<br>Line 480-482

*The authors created the SRQR by searching the literature to identify guidelines, reporting standards, and critical appraisal criteria for qualitative research; reviewing the reference lists of retrieved sources; and contacting experts to gain feedback. The SRQR aims to improve the transparency of all aspects of qualitative research by providing clear standards for reporting qualitative research.

**The rationale should briefly discuss the justification for choosing that theory, approach, method, or technique rather than other options available, the assumptions and limitations implicit in those choices, and how those choices influence study conclusions and transferability. As appropriate, the rationale for several items might be discussed together.

Reference:

O'Brien BC, Harris IB, Beckman TJ, Reed DA, Cook DA. **Standards for reporting qualitative research: a synthesis of recommendations.** *Academic Medicine*, Vol. 89, No. 9 / Sept 2014
DOI: 10.1097/ACM.0000000000000388
