# Supplementary material for: Learning for doctor-to-doctor collaboration: a qualitative study exploring the experiences of residents and supervisors with intraprofessional workplace learning in complex tertiary care
Source: BMC Med Educ. 2023 Jun 27;23:478. doi: 10.1186/s12909-023-04363-5 (PMC10303293; doi:10.1186/s12909-023-04363-5)
Supplement: Supplementary file 2 — Supplementary Material 2 [file 12909_2023_4363_MOESM2_ESM.pdf]

Additional file 2: interview guide individual interview

Learning for doctor-to-doctor collaboration: a qualitative study exploring the experiences of residents and supervisors with intraprofessional workplace learning in complex tertiary care

BMC Medical Education

Lara Teheux, Hanna Wollaars, Jos M.T. Draaisma, Ester H.A.J. Coolen, Wietske Kuijer-Siebelink, Janiëlle A.E.M. van der Velden.

Corresponding author
Lara Teheux, MD, PhD-student, Department of Pediatrics, Amalia Children's Hospital, Radboud University Medical Center, Nijmegen, The Netherlands.
E-mail: lara.teheux@radboudumc.nl

As you have just read, the Radboudumc Amalia Children’s Hospital aims to realize an intraprofessional learning environment for residents that provides residents from various continued medical training programs with joint instruction across the boundaries of their own specialties. We define intraprofessional collaboration as the collaboration between healthcare professionals of two or more disciplines within the same profession, such as pediatricians and surgeons. The two steps in this project are 1) recognizing and acknowledging existing, intraprofessional training occasions, and 2) developing intraprofessional education with a clear link to the workplace.

This interview primarily concerns step one. The questions are aimed at acquiring an insight into the possibilities you see for (the further development of) intraprofessional collaboration and training, and the opportunities and pitfalls you identify concerning this topic.

1. You are familiar with the Amalia Children’s Hospital’s vision and plans with regard to intraprofessional collaboration, learning and training. How do you feel about this matter?
   What do you find appealing?
   Or what don’t you find appealing?

2. What instances of intraprofessional education and collaboration do you observe already in current practice? Both in education as in the workplace.
    - What about these is going well?
    - What about these requires attention?

3. What value do these intraprofessional training moments and collaboration have for you?
    - What positive impact does it have for you/residents?
    - What negative consequences does it have for you/residents?

4. What potential additional opportunities for intraprofessional learning do you see in your education that are currently underutilized?
    - What are factors that might assist in utilizing these?

5. What ideas do you have to further shape intraprofessional learning and working in practice/in the workplace?
    - What are facilitative factors?

6. What barriers do you observe for the further development of intraprofessional education?

7. What barriers do you observe for the further development of intraprofessional working and workplace learning?

8. What advice do you have for the Amalia Hospital that could contribute to the success of intraprofessional collaboration, learning and teaching?
    - What are your needs in this respect?
