# Supplementary material for: Learning for doctor-to-doctor collaboration: a qualitative study exploring the experiences of residents and supervisors with intraprofessional workplace learning in complex tertiary care
Source: BMC Med Educ. 2023 Jun 27;23:478. doi: 10.1186/s12909-023-04363-5 (PMC10303293; doi:10.1186/s12909-023-04363-5)
Supplement: Supplementary file 3 — Supplementary Material 3 [file 12909_2023_4363_MOESM3_ESM.pdf]

Additional file 3: guiding questions focus group interview

Learning for doctor-to-doctor collaboration: a qualitative study exploring the experiences of residents and supervisors with intraprofessional workplace learning in complex tertiary care

BMC Medical Education

Lara Teheux, Hanna Wollaars, Jos M.T. Draaisma, Ester H.A.J. Coolen, Wietske Kuijer-Siebelink, Janiëlle A.E.M. van der Velden.

Corresponding author

Lara Teheux, MD, PhD-student, Department of Pediatrics, Amalia Children's Hospital, Radboud University Medical Center, Nijmegen, The Netherlands.

E-mail: lara.teheux@radboudumc.nl

The researcher briefly presents the themes of the individual interviews about intraprofessional learning in residency training.

What examples stand out positively for you? What is your experience?

What would you like to build upon or explore or apply further?

In what ways could daily work be deployed more emphatically to encourage intraprofessional learning in residents?

What suggestions and experiences do you have to minimalize the impact of the mentioned barriers?

What support can help in this regard?
